# Are Measurement Instruments Responsive to Assess Acute Responses to Load in High-Level Youth Soccer Players?

**DOI:** 10.3389/fspor.2022.879858

**Published:** 2022-07-01

**Authors:** Ludwig Ruf, Barry Drust, Paul Ehmann, Sabrina Skorski, Tim Meyer

**Affiliations:** ^1^Institute of Sports and Preventive Medicine, Saarland University, Saarbrücken, Germany; ^2^TSG ResearchLab gGmbH, Zuzenhausen, Germany; ^3^TSG 1899 Hoffenheim, Zuzenhausen, Germany; ^4^School of Sport, Exercise and Rehabilitation Sciences, University of Birmingham, Birmingham, United Kingdom

**Keywords:** training load, monitoring, fatigue, adolescence, responsiveness

## Abstract

**Purpose:**

The aim of this study was to assess the short-term responsiveness of measurement instruments aiming at quantifying the acute psycho-physiological response to load in high-level adolescent soccer players.

**Methods:**

Data were collected from 16 high-level male youth soccer players from the Under 15 age group. Players were assessed on two occasions during the week: after 2 days of load accumulation (“high load”) and after at least 48 h of rest. Measurements consisted of the Short Recovery and Stress Scale (SRSS), a countermovement jump (CMJ) and a sub-maximal run to assess exercise heart-rate (HRex) and heart-rate recovery (HRR60s). Training load was quantified using total distance and high-speed running distance to express external and sRPE training load to express internal load. It was expected that good instruments can distinguish reliably between high load and rest.

**Results:**

Odd ratios (0.74–1.73) of rating one unit higher or lower were very low for athlete-reported ratings of stress and recovery of the SRSS. Standardized mean high load vs. rest differences for CMJ parameters were trivial to small (−0.31 to 0.34). The degree of evidence against the null hypothesis that changes are interchangeable ranged from *p* = 0.04 to *p* = 0.83. Moderate changes were observed for HRex (−0.62; 90% Cl −0.78 to −0.47; *p* = 3.24 × 10^−9^), while small changes were evident for HRR60s (0.45; 90% Cl 0.08–0.80; *p* = 0.04). Only small to moderate repeated-measures correlations were found between the accumulation of load and acute responses across all measurement instruments. The strongest relationships were observed between HRex and total distance (rm-r = −0.48; 90% Cl −0.76 to −0.25).

**Conclusion:**

Results suggest that most of the investigated measurement instruments to assess acute psycho-physiological responses in adolescent soccer players have limited short-term responsiveness. This questions their potential usefulness to detect meaningful changes and manage subsequent training load and program adequate recovery.

## Introduction

Athlete monitoring frameworks are widely implemented and considered as important aspects of the training process to maximize sports performance and health, and in turn minimize injury risk, in professional senior and youth soccer environments. The training process is the systematic repetition of physical training comprising external, i.e., the prescribed quantity and intensity of the training plan, and internal load, i.e., psycho-physiological stress experienced by an athlete during the training session and subsequent associated responses (Jeffries et al., 2020). Conceptually, sports performance can be improved when loading an athlete's biological system to induce adaptive responses. However, stressing the athlete's biological systems has to be balanced with appropriate recovery periods to allow for positive adaptations to occur. This highlights the importance of both measuring and managing training load and the associated acute responses (Impellizzeri et al., 2019).

Response to load is a broader construct encompassing multiple domains including cardiorespiratory, metabolic, neuromuscular, endocrine, musculoskeletal as well as overall functional output (McLaren et al., 2021). Monitoring acute responses to load is commonly used by coaches and practitioners to decide about subsequent training load, evaluate the effectiveness of the training program and prescribe adequate recovery (Weston, 2018; Salter et al., 2021). This entails a holistic approach involving constructs that are not directly measurable, instead they have to be quantified *via* surrogate measurement instruments. As such, several measurement instruments are typically selected by coaches and practitioners based on contextual considerations such as specificity to the sport, time efficiency, scalability to large groups, availability, and theoretical aspects such as relevant measurement properties (Coutts, 2014; Robertson et al., 2017; Thorpe et al., 2017; Starling and Lambert, 2018).

Before confidently adopting measurement instruments to assess acute responses to load, several measurement properties need to be assessed and critically appraised to understand whether their quality supports their implementation by practitioners and scientists. Little attention, however, has been given to the responsiveness of parameter and measurements to assess realistically occurring acute responses to load in youth soccer. Responsiveness is defined by the COSMIN panel as the ability of a parameter to detect change over time in the construct to be measured (Mokkink et al., 2010). Responsiveness has been considered as the most important property of measurement instruments (Terwee et al., 2003). Several approaches exist to assess the responsiveness of a given measurement instrument. However, as no gold-standard measurement instruments exist to date to quantify the construct of acute responses to load, a construct-based approach has to be adopted in applied sport science (Vet et al., 2011). That is, repeated measurements are required in which changes in the constructs of interest, i.e., acute responses to load, are expected to occur for some proportion of the participants. The assessment of acute responses after soccer matches or intense training sessions might represent a suitable setting whereby large changes in acute responses are expected for players who accumulated more external and internal loads. The stronger the relationship between both constructs, i.e., accumulated load and acute responses and, the greater the responsiveness for the given measurement instrument.

Few studies have investigated the responsiveness of measurement instruments to assess acute responses to load in professional senior athletes and even fewer in youth populations. Various different methods and measurement instruments have been investigated within late adolescent team sport athletes (Malone et al., 2015b; Noon et al., 2015; Pelka et al., 2018; Sawczuk et al., 2018b; Fitzpatrick et al., 2019). Objective measurement instruments such as squat jump and countermovement jump (CMJ) height appear to lack responsiveness to short periods of intensified load accumulation (Fitzpatrick et al., 2019). No studies, however, have investigated whether parameters related to the kinematics and kinetics of a CMJ are acutely affected after the accumulation of training loads. In contrast, changes in athlete-reported measurements related to the constructs of fatigue and recovery have been observed in response to intense training sessions and matches (Saw et al., 2016). However, previous research was mainly carried out with athletes during the late adolescence period, ranging from 16 to 19 years of age, or adults. As youth athletes mature from early to late adolescence they experience larger exercise-induced physiological responses to a given load due to changes in muscle mass, fiber type composition, energy metabolism and voluntary activation level occurring during adolescence (Beunen and Malina, 2007; Ratel and Martin, 2015). This is due to the large associated hormonal changes of the hypothalamic-pituitary axes occurring as adolescents enter the phase of peak height velocity directly regulating the maturation of specific structures and tissues (Beunen et al., 2006). As such responses to load may be maturity-dependent in a way that pre-pubertal children show the smallest responses to load followed by adolescent and adult athletes (Ratel and Williams, 2017). Similarly, youth athletes have to cope with non-spot related stressors such as academic and social issues. Periods of high academic stress potentially superimposes the psycho-physiological responses to load and subsequent physiological adaptation given the lack of adequate coping strategies of youth athletes (Cosh and Tully, 2015). Coaches and practitioners need to be aware of such influencing factors when interpreting psycho-physiological changes to load. This highlights the unique and challenging environment and characteristics of youth athletes aiming to assess responses to load (Scantlebury et al., 2020). Therefore, acute changes to load in athlete-reported measurement instruments within less mature athletes may be smaller, reducing the responsiveness of this measurement instrument.

Therefore, this study aimed to assess the short-term responsiveness of measurement instruments that quantify the acute psycho-physiological response to load in high-level adolescent soccer player. We hypothesize that a few days of accumulated load in adolescent soccer players will (be associated with a) change countermovement jump variables, psychological variables (as measured by the constructs within the Short Recovery and Stress Scale), and heat rate variables derived from a 4-min sub-maximal shuttle run. This information potentially provides practitioners working with youth soccer players additional insight regarding the usefulness of commonly used measurement instruments to assess acute responses to load.

## Methods

### Participants

Data were collected from 16 male youth soccer players (chronological age: 14.4 ± 0.3 years, skeletal age: 15.4 ± 1.1 years, 96 ± 3% of predicted adult height, post-pubertal [determined using the BAUS™ system (SonicBone Medical Ltd., Israel) (Ruf et al., 2021a)], standing height: 170.0 ± 6.6 cm, body mass: 62.8 ± 9.0 kg) from the Under 15 age group of one professional German youth academy. Testing was conducted during the first half of the 2020/21 season. All participants were medically cleared to participate in formalized soccer practice. Prior to the commencement of the study, all participants were informed about the aims, procedures, and risks of the investigation. Parental or guardian consent for all participants involved in this study was obtained. The study was approved by the institutional ethics committee and was conducted according to the Declaration of Helsinki.

### Design

**Figure 1:**
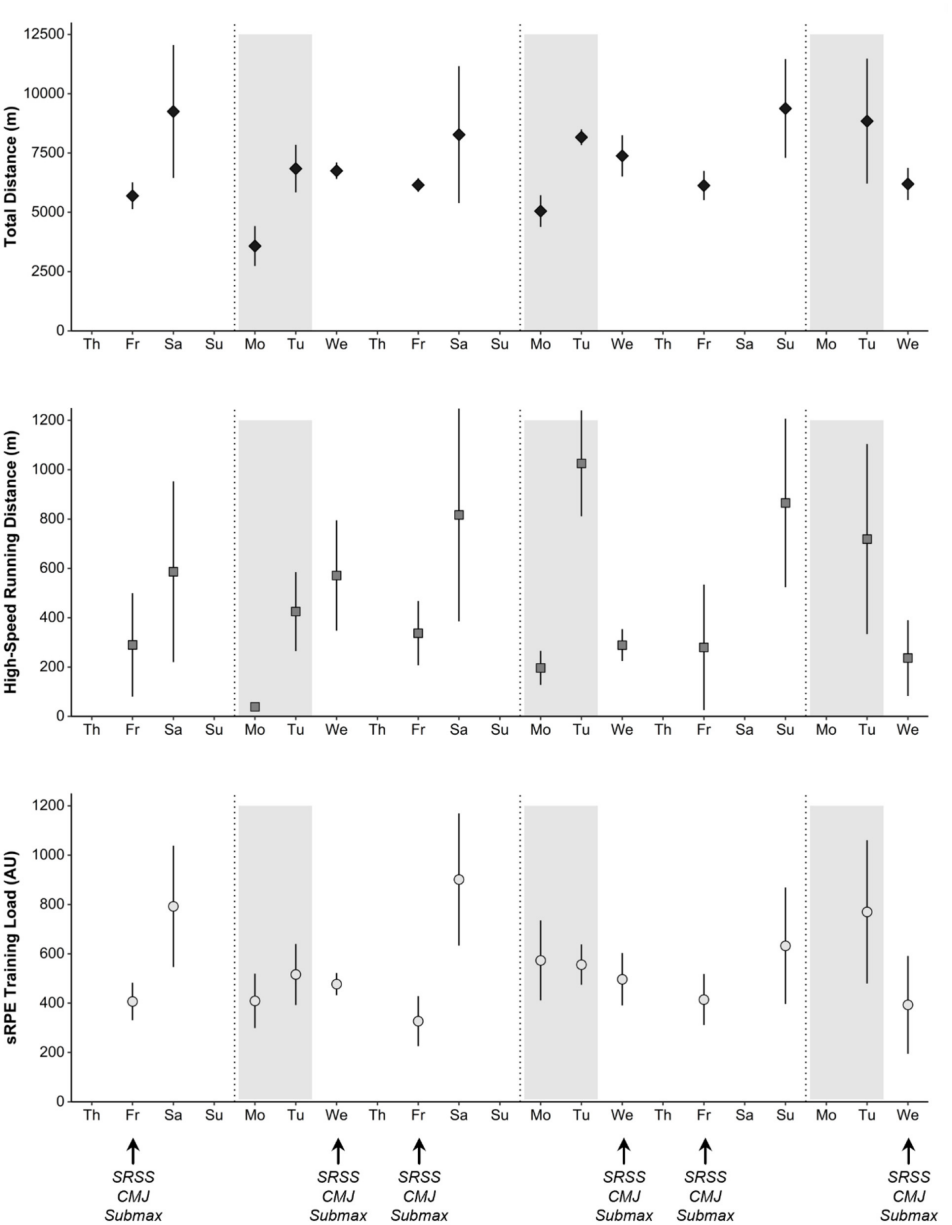
Mean ± SD of daily training load for sRPE Training Load (upper panel), Total Distance (middle panel) and High-Speed Distance (lower panel) across the study period. The gray area represents the 2 days of load accumulation (see methods). Abbreviations: SRSS: Short Recovery and Stress Scale; CMJ: countermovement jump; Submax: sub-maximal run.

The study comprised a 4-week observational period during the regular in-season period (September–October 2020). A schematic overview is illustrated in Figure 1. Players were tested on Friday and Wednesday of the subsequent week before the regular training session (~4:30–5:30 pm). In total, six testing days were included in the final analysis (three on Wednesday and three on Friday). Testing on Wednesday was scheduled after 2 days of load accumulation. In contrast, testing on Friday was conducted after at least 48 h of rest. These testing days were chosen to represent contrasting conditions to maximize the chance for the detection of substantial differences by the measurement instruments assessing the acute responses to the accumulation of high load (Wednesday) vs. rest (Friday). Upon arrival, players filled out the subjective recovery-stress status questionnaire (Short Recovery and Stress Scale, SRSS), performed two CMJs on a force plate, and a sub-maximal run as part of the team training warm-up to calculate heart rate responses during and after the run.

#### Anthropometry and Maturation Status

Participant's standing height, body mass and chronological age were measured during the first week of the study. Standing height (±0.1 cm, seca 213 portable stadiometer, Seca, Hamburg, Germany) and body mass (±0.1 kg, seca 813, calibrated digital scale, Seca, Hamburg, Germany) were measured according to standardized ISAK measurement techniques (Stewart et al., 2011).

#### Short Recovery-Stress Scale

On testing days, participants completed the German version of the modified Short Recovery and Stress Scale (SRSS) for children/adolescents (SRSS (Kellmann and Kölling, 2020) upon arriving at the training facilities. The questionnaire consists of four items each for the Short Recovery Scale (i.e., Physical Performance Capability, Mental Performance Capability, Emotional Balance, Overall Recovery) and the Short Stress Scale (i.e., Muscular Stress, Lack of Activation, Negative Emotional State, Overall Stress). For each item, a sentence was provided to describe it complementing the four descriptive adjectives. Items were scored on a seven-point Likert scale with single point increments, ranging from does not apply at all (0) to fully applies (6). Structural, construct and cross-cultural validity and strong internal consistency have been reported for both the recovery (α = 0.73–0.78) and stress scale (α = 0.72–0.80) in youth and adolescent athletes (Kölling et al., 2019). Reliability of the selected parameters were assessed in a recent short-term between-days reliability study conducted in youth soccer players (Ruf et al., 2021b). Coefficients of variations ranged between 18% (Mental Performance Capability) and 52% (Muscular Stress) for the post peak height-velocity group.

#### Countermovement Jump (CMJ)

Following a standardized dynamic warm-up consisting of dynamic stretching and sub-maximal jumping, participants performed two CMJs. Participants were required to keep their hands held in place on the hips and instructed to jump as high as possible. CMJ depth and stance were self-selected by the participants. Jumps were performed on a portable dual force plate recording simultaneously vertical forces at 1,000 Hz (GEN2 Dual Force Plate, Hawkin Dynamics, Inc., Westbrook, Maine, USA). Left-side and right-side vertical forces were summed to single force-time curves for analysis. Data were collected and stored using the proprietary application (Hawkin Capture, version 7.1.1) on a tablet (Samsung Galaxy Tab A, model number SM-T510, Samsung Electronics Co., Ltd., Suwon, South Korea) connected *via* Bluetooth to the force plates. Force-time data analysis was done using the proprietary software, which followed the methods previously described by Lake et al. (2018). The following variables were derived from the force-time data of the CMJ: jump height (JH, cm), reactive strength index modified (RSImod, m.s^−1^), eccentric rate of force development (RFD, N.s^−1^), eccentric impulse (EccI, N.s), concentric impulse (ConI, N.s.), average eccentric velocity (EccV, m.s^−1^), average concentric velocity (ConV, m.s^−1^), force at zero velocity (F@0V, N), duration of eccentric phase (DurEcc, ms), duration of concentric phase (DurCon, ms), countermovement depth (CMD, cm) (see Supplementary File, Table S1 for detailed description). Variables from that CMJ with the highest velocity at take-off were used for subsequent analysis. Reliability of the selected parameters were assessed in a recent short-term between-days reliability study conducted in youth soccer players (Ruf et al., 2021b). Coefficients of variations ranged between 3.7% (ConV) and 30.5% (RDF) with most parameters showing coefficients of variations smaller than 10% for the post peak-height velocity group.

#### Sub-maximal Run

A 4-min continuous shuttle run followed by a 1-min passive (standing) recovery period was performed outdoor at the start of the training session on an artificial turf. Recent research indicated that the assessment of heart rate during sub-maximal runs fluctuates in relation to short-term accumulation of load making this measurement instrument a viable option to monitor cardio-respiratory responses to load (Schneider et al., 2020). All players were tested at the same time. Shuttle distances, times and corresponding average speeds were as follows: 50 m in 15 s at 12.0 km.h^−1^. After the 4-min continuous shuttle run, players were required to remain in a stationary standing position avoiding any movement. Heart rate was recorded continuously at 1 Hz (Polar Team Pro, Polar Electro Oy, Kempele, Finland) and raw data were subsequently downloaded from manufacturers' proprietary software (Team Pro, version 2.0.4, Polar Electro Oy, Kempele, Finland). Mean heart rate during the final 30 s (HRex, beats.min^−1^) of the 4-min continuous shuttle run was computed (Rabbani et al., 2017). Heart rate recovery was calculated as the absolute difference between HRex and heart rate after the 1-min recovery period (HRR60s, beats.min^−1^). Reliability of the selected parameters were assessed in a recent short-term between-days reliability study conducted in youth soccer players (Ruf et al., 2021b). Coefficients of variations were 1.7% for HRex and 12.8% for the post peak-height velocity group.

#### Training Load Quantification

External training load was monitored using a global positioning system (GPS). During each training session athletes wore a GPS device (Polar Team Pro; Polar Electro Oy, Kempele, Finland) sampling at 10 Hz. The device was worn on a custom chest belt and athletes were assigned the same device throughout the study period. The following external load variables were selected: total distance (TD, *m*), high-speed running distance (HSRD, *m* > 4.7 m.s^−1^). Internal training load was determined by multiplying the session-rating of perceived exertion (sRPE) by the session duration in minutes to derive sRPE training load (Foster et al., 2001). Following each session, athletes individually reported their sRPE using Borg's modified CR10 scale via a bespoke smartphone application. Ratings were reported at 8 p.m., ~15–30 min following the end of the session. In youth team sports, sRPE has been shown to possess acceptable construct validity as a measure of exercise intensity and internal load (Foster et al., 2021).

### Statistical Analyses

Data are presented either as mean with standard deviations (SD) or 90% confidence intervals (90% CI). Items of the SRSS were treated as ordinal variables and analyzed *via* ordered logistic regression models [*MASS* package (Venables et al., 2002)]. Linear mixed models (*nlme* package (Pinheiro et al., 2019) assessed the changes in CMJ (continuous variables: JH, RSImod, RFD, N.s^−1^, EccI, N.s, ConI, N.s., EccV, m.s^−1^, ConV, m.s^−1^, F@0V, N, DurEcc, DurCon, CMD), and submaximal running heart rate variables (continuous variables: HRex, HRR60s) over the training week [categorical factor, 2 levels: Rest (Friday), High Load (Wednesday)]. Correlated random effects were fit by specifying a random intercept for athlete ID and a random slope for time (i.e., Rest and High Load) to account for the individual player difference over the training week. Autocorrelation was specified *via* the exponential variance-covariance matrix and weights were specified *via* a constant variance function structure to allow for heterogenous within-subject variances by time (i.e., Rest and High Load).

Changes in the measurement instruments were also assessed by calculating standardized mean differences (SMD, based on Cohen's d's effect size principle using pooled SD). In addition, repeated-measures correlations (rm-r) between changes in the measurement instruments and indicators of external and internal load were computed [*rmcorr* package (Bakdash and Marusich, 2020)]. Bootstrapping (with 10,000 resamples) was used to derive CIs for SMD and rm-r. Threshold values for SMD were as follows: ≤ 0.2 (trivial), >0.2–0.6 (small), >0.6–1.2 (moderate), and >1.2 (large). Threshold values for rm-r were as follows: ≤ 0.10 (trivial), >0.10–0.30 (small), >0.30–0.50 (moderate), >0.50–0.70 (very large), and >0.70–1.00 (excellent). All analysis were performed in Rstudio (version 1.2.5033, RStudio Inc.) with a more detailed outline of the statistical analysis presented in the Supplementary Material.

## Results

Descriptive data (mean ± SD) for total distance, high-speed running distance and sRPE training load across the data collection period were 10,461 ± 2,644 m, 821 ± 420 m and 921 ± 274 AU. Daily and accumulated training loads are summarized in Figure 1 and Table 1.

**Table 1:** Mean ± SD of daily and accumulated training load across the data collection period.

**Week**	**Loading pattern**	**Total distance (m)**		**High-speed running distance (m)**		**sRPE training load (AU)**	
		***Day 1 Monday***	***Day 2*** ***Tuesday***	***Day 1 Monday***	***Day 2 Tuesday***	***Day 1 Monday***	***Day 2 Tuesday***
Week 1	Daily training load	3,578 ± 843	6,841 ± 1,003	39 ± 52	425 ± 160	409 ± 110	516 ± 124
	Accumulated training load	9,688 ± 1,071		455 ± 126		925 ± 201	
Week 2	Daily training load	5,050 ± 666	8,164 ± 333	196 ± 69	1,025 ± 214	573 ± 162	556 ± 82
	Accumulated training load	12,793 ± 1,746		1,205 ± 262		1,081 ± 222	
Week 3	Daily training load	na	8,845 ± 2,635	na	719 ± 386	na	770 ± 290
	Accumulated training load	8,845 ± 2,635		719 ± 386		770 ± 290	

Descriptive statistics, results of the linear mixed models and standardized mean differences are summarized in Table 2 for the items of the SRSS, in Table 3 for parameters of the CMJ and Table 4 for parameters of the submaximal run. The observed mean changes in the eight parameters of the SRSS ranged from −0.23 to 0.41 (90% confidence intervals −0.51 to 0.74) the degree of evidence against the null hypothesis that the changes are interchangeable ranged from *p* = 0.20 to *p* = 0.98. Trivial to small standardized changes were evident across the parameters of the CMJ. The degree of evidence against the null hypothesis that changes are interchangeable ranged from *p* = 0.04 to *p* = 0.83, indicating large confidence intervals and in turn a wide range of plausible true effects. A moderate (90% confidence interval of [−3.4 to −2.2], *p* = 5.56 × 10^−10^) decrease in HRex and small (90% confidence interval of [0.5 to 3.8], *p* = 0.02) increase in HRR60s was observed across the two testing assessments.

**Table 2:** Descriptive statistics, and odds ratio derived from the ordered logistic regression models of the Short Recovery and Stress Scale (SRSS) (number of observations: 68).

**Variable**	**Rest**	**High load**	**Odds ratio (90% CI)**	***p*-value**
	**Frequency count of each rating**	**Frequency count of each rating**		
**Short Recovery Scale**				
Physical Performance Capability (AU)	0: 0	0: 0	0.74	0.49
	1: 0	1: 1	(0.35–1.54)	
	2: 3	2: 1		
	3: 2	3: 8		
	4: 7	4: 4		
	5: 14	5: 13		
	6: 8	6: 7		
Mental Performance Capability (AU)	0: 0	0: 0	0.83	0.68
	1: 0	1: 0	(0.40–1.73)	
	2: 1	2: 2		
	3: 5	3: 7		
	4: 8	4: 5		
	5: 11	5: 12		
	6: 9	6: 8		
Emotional Balance (AU)	0: 0	0: 0	0.93	0.87
	1: 0	1: 0	(0.45–1.93)	
	2: 2	2: 3		
	3: 2	3: 3		
	4: 9	4: 7		
	5: 10	5: 10		
	6: 11	6: 11		
Overall Recovery (AU)	0: 0	0: 0	0.99	0.98
	1: 1	1: 0	(0.48–2.03)	
	2: 2	2: 6		
	3: 5	3: 3		
	4: 12	4: 7		
	5: 6	5: 13		
	6: 8	6: 5		
**Short Stress Scale**				
Muscular Stress (AU)	0: 8	0: 7	1.18	0.71
	1: 10	1: 9	(0.57–2.42)	
	2: 6	2: 8		
	3: 5	3: 5		
	4: 4	4: 2		
	5: 1	5: 3		
	6: 0	6: 0		
Lack of Activation (AU)	0: 17	0: 13	1.36	0.50
	1: 8	1: 14	(0.64–2.88)	
	2: 7	2: 2		
	3: 2	3: 2		
	4: 0	4: 2		
	5: 0	5: 0		
	6: 0	6: 1		
Negative Emotional State (AU)	0: 24	0: 20	1.80	0.25
	1: 9	1: 10	(0.77–4.18)	
	2: 0	2: 4		
	3: 1	3: 0		
	4: 0	4: 0		
	5: 0	5: 0		
	6: 0	6: 0		
Overall Stress (AU)	0: 21	0: 16	1.83	0.20
	1: 4	1: 6	(0.83–3.98)	
	2: 6	2: 6		
	3: 3	3: 3		
	4: 0	4: 3		
	5: 0	5: 0		
	6: 0	6: 0		

*Note: Rest refers to the testing scheduled on Friday, which was conducted after at least 48 h of rest; high load refers to the testing scheduled on Wednesday, which was conducted after 2 days of load accumulation; mean change refers to the absolute difference between high load and rest; CI, confidence interval*.

**Table 3:** Descriptive statistics, standardized mean differences and results of the linear mixed models for parameters of the countermovement jump (CMJ) (number of players: 16; number of observations: 68; df: 51).

	**Jump height (cm)**	**Reactive strength index modified (m.s^**−1**^)**	**Eccentric rate of force development (N.s^**−1**^.kg^**−1**^)**	**Eccentric impulse (N.s.kg^**−1**^)**	**Concentric impulse (N.s.kg^**−1**^)**	**Eccentric velocity (m.s^**−1**^)**	**Concentric velocity (m.s^**−1**^)**	**Force at zero velocity (N.kg^**−1**^)**	**Duration of eccentric phase (ms)**	**Duration of concentric phase (ms)**	**Countermovement depth (cm)**
**Predictors**											
Rest Mean ± SD	33.2 ± 4.2	0.40 ± 0.07	85.7 ± 35.4	2.7 ± 0.5	5.0 ± 0.5	−0.72 ± 0.14	1.52 ± 0.12	22.8 ± 2.7	168 ± 44.4	250 ± 47.1	−28.3 ± 5.7
High Load mean ± SD	32.9 ± 3.9	0.39 ± 0.07	74.6 ± 29.7	2.8 ± 0.4	5.0 ± 0.5	−0.73 ± 0.16	1.50 ± 0.11	22.1 ± 2.4	176 ± 35.6	254 ± 43.3	−29.1 ± 6.0
Intercept (90% CI)	0.33 (0.31 to 0.35	0.40 (0.37 to 0.42)	84.9 (72.7 to 97.2)	2.7 (2.5 to 2.9)	5.0 (4.8 to 5.2)	−0.72 (−0.78 to −0.66)	1.52 (1.47 to 1.57)	22.8 (21.7 to 23.9)	170 (152 to 187)	250 (231 to 270)	−28.3 (−30.8 to −25.8)
Mean change High Load minus Rest (90% CI)	−0.25 (−0.75 to 0.28)	−0.10 (−0.03 to 0.01)	−11.1 (−23.9 to 1.4)	0.1 (−0.0 to 0.2)	0.0 (−0.0 to 0.1)	0.00 (−0.03 to 0.03)	−0.02 (−0.04 to 0.00)	−0.7 (−1.7 to 0.1)	8 (−4 to 21)	4 (−3 to 11)	−0.76 (−1.7 to 0.2)
*p*-value	*p* = 0.37	*p* = 0.13	*p* = 0.07	*p* = 0.15	*p* = 0.48	*p* = 0.56	*p* = 0.12	*p* = 0.08	*p* = 0.28	*p* = 0.36	*p* = 0.16
Standardized mean difference (90% CI)	−0.06 (−0.18 to 0.07)	−0.14 (−0.38 to 0.09)	−0.31 (−0.59 to −0.02)	0.19 (−0.02 to 0.37)	0.06 (−0.08 to 0.19)	−0.03 (−0.19 to 0.11)	−0.16 (−0.35 to 0.05)	−0.28 (−0.58 to 0.00)	0.18 (−0.05 to 0.45)	0.08 (−0.08 to 0.24)	−0.13 (−0.27 to 0.01)
**Random Effects**											
SD residual (90% CI)	1.27 (0.96 to 1.67)	0.06 (0.03 to 0.10)	17.3 (14.5 to 21.2)	0.23 (0.16 to 0.31)	0.19 (0.15 to 0.25)	0.10 (0.06 to 0.17)	0.06 (0.04 to 0.07)	2.3 (1.0 to 5.2)	44 (8 to 225)	19 (15 to 24)	2.3 (1.9 to 2.7)
Between-subject-SD τ00 (90% CI)	4.1 (3.0 to 5.6)	0.05 (0.02 to 0.12)	30.2 (21.7 to 43.2)	0.46 (0.33 to 0.61)	0.47 (0.36 to 0.67)	0.12 (0.06 to 0.19)	0.12 (0.08 to 0.16)	1.6 (0.3 to 8.3)	38 (23 to 50)	45 (32 to 60)	5.4 (4.0 to 7.6)
Random-slope-SD τ11 (90% CI)	0.4 (0.02 to 0.8)	0.02 (0.006 to 0.07)	24.8 (14.8 to 38.0)	0.18 (0.10 to 0.30)	0.02 (0.005 to 0.14)	0.02 (0.005 to 0.10)	0.03 (0.06 to 0.11)	1.7 (1.2 to 2.6)	20 (6 to 32)	4 (1 to 25)	0.41 (0.08 to 1.8)
Phi parameter (90% CI)	0.09 (−0.46 to 0.59)	0.63 (0.06 to 0.89)	−0.04 (−0.39 to 0.33)	0.36 (−0.12 to 0.71)	0.08 (−0.28 to 0.41)	0.83 (0.60 to 0.93)	0.11 (−0.31 to 0.50)	0.79 (0.10 to 0.96)	0.86 (−0.44 to 0.99)	−0.05 (−0.36 to 0.28)	0.04 (−0.26 to 0.34)

*Note: Rest refers to the testing scheduled on Friday, which was conducted after at least 48 h of rest; high load refers to the testing scheduled on Wednesday, which was conducted after 2 days of load accumulation; mean change refers to the absolute difference between high load and rest; CI, confidence interval; SE, standard error; df, degrees of freedom*.

**Table 4:** Descriptive statistics, standardized mean differences and results of the linear mixed models for parameters of the submaximal run (number of players: 16; number of observations: 68; df: 49).

	**HRex (%)**	**HRR60s (%)**
**Predictors**		
Rest Mean ± SD	87.6 ± 4.4	23.3 ± 4.5
High load Mean ± SD	84.8 ± 4.7	25.4 ± 5.4
Intercept (90% CI)	87.6 (85.8 to 89.4)	23.4 (21.7 to 25.1)
Mean change high load minus rest (90% CI)	−2.8 (−3.4 to −2.2)	2.1 (0.5 to 3.8)
*p*-value	*p* = 5.56 × 10^−10^	*p* = 0.02
Standardized mean difference (90% CI)	−0.62 (−0.78 to −0.47)	0.45 (0.08 to 0.80)
**Random effects**		
SD residual (90% CI)	1.5 (1.3 to 1.7)	3.7 (3.2 to 4.4)
Between-subject-SD τ00 (90% CI)	4.1 (2.9 to 5.5)	2.8 (1.5 to 4.7)
Random-slope-SD τ11 (90% CI)	0.3 (0.003 to 0.9)	0.7 (0.001 to 3.1)
Phi parameter (90% CI)	−0.10 (0.40 to 0.22)	−0.02 (−0.38 to 0.34)

*Note: Rest refers to the testing scheduled on Friday, which was conducted after at least 48 h of rest; high load refers to the testing scheduled on Wednesday, which was conducted after 2 days of load accumulation; mean change refers to the absolute difference between high load and rest; CI, confidence interval; SE, standard error; df, degrees of freedom*.

**Figure 2:**
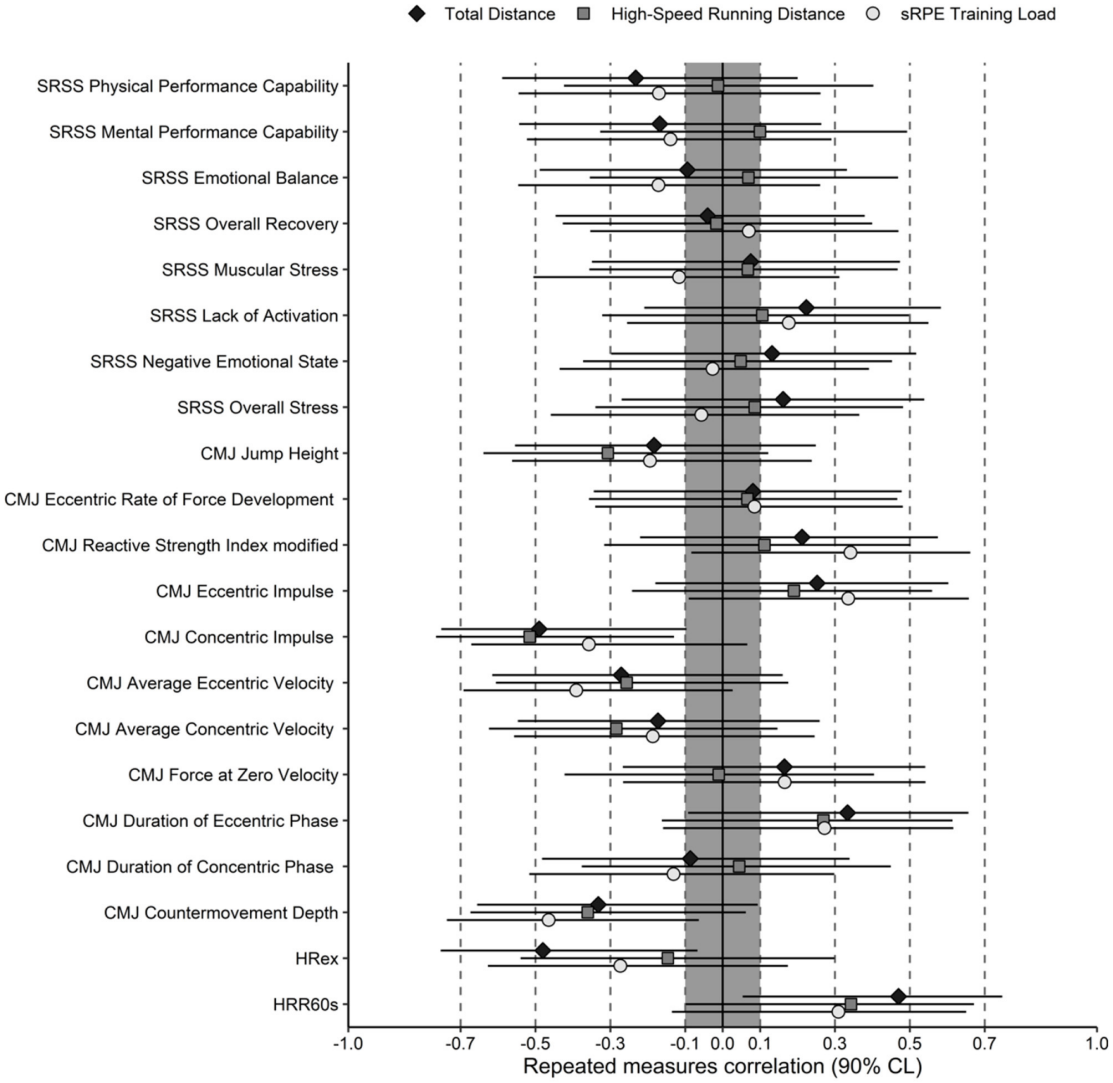
Repeated measures correlation (90% CI) between changes in measurement instruments and training load indicators total distance, high-speed running distance and sRPE training load. The gray area represents trivial correlations.

Repeated-measures correlations between changes in the measurement instruments and indicators of external and internal load revealed small to moderate associations (Figure 2). The strongest relationships were observed between HRex and total distance (rm-r = −0.48; 90% Cl −0.76 to −0.25), HRR60s and total distance (rm-r = 0.47; 90% Cl 0.06 to 0.74), and CMJ concentric impulse and sRPE training load (rm-r = −0.47; 90% Cl −0.74 to −0.06).

## Discussion

The aim of the current investigation was to determine short-term responsiveness of measurement instruments aiming to quantify the acute psycho-physiological response to load in high-level adolescent soccer players. The results of the study demonstrated that (1) magnitudes of changes were trivial to small for athlete-reported ratings of stress and recovery (<1 AU) and CMJ parameters (0–13%) and smaller than the typically observed day-to-day variability (~1 AU for athlete-reported rating of stress and recovery; 3.7–30.5% for CMJ parameter), (2) a moderate change with a narrow range for plausible true effects as inferred by the confidence limits, exceeding the typically observed day-to-day variability, was observed for HRex, while a small change was evident for HRR60s, which was however smaller than the typically observed day-to-day variability, and (3) small to moderate relationships were evident between the accumulation of load and acute responses. Collectively, these findings highlight the poor responsiveness of most investigated parameters questioning their potential utility to monitor psycho-physiological responses to load that are beyond the typically observed day-to-day variability in adolescent soccer players. As such, using these measurement instruments to make conclusions on an athlete's psycho-physiological status and in turn to adjust subsequent training loads cannot be recommended.

The current study is one of the first to investigate the relationship between training load indicators and acute psycho-physiological responses in adolescent soccer players. Findings of this study support previous studies that identified a limited responsiveness of measurement instruments that aim to quantify the acute psycho-physiological response to load in adolescent athletes (Sawczuk et al., 2018b; Fitzpatrick et al., 2019). This is in contrast to a recent systematic review suggesting that athlete-reported measurement instruments have a better responsiveness to acute training load than objective measures in adults (Saw et al., 2016). In addition, adolescent athletes experience non-sport related stressors such as academic and social pressures potentially impacting the perception of recovery and stress. However, changes in all items of the SRSS were <1 point which is less than the typical day-to-day variability observed in youth soccer players (typical error of measurement: ~1 point), and less than the minimal detectable change which is one point on a seven-point ordered scale since that is the minimal possible measurement unit (Ruf et al., 2021b), accompanied with low odds of players rating one unit higher or lower on the scale. Similarly, correlations between training load and items of the SRSS were ranged between trivial to small. Discrepancies in these findings can potentially be attributed to the inherent differences between the two populations. Adolescent athletes have a unique set of psychological and physiological characteristics and environmental circumstances. As youth athletes mature toward adulthood the substantial physiological changes create larger exercise-induced responses to load (Ratel and Martin, 2015; Ratel and Williams, 2017). However, given that adolescence marks a critical period of emotional and cognitive development with the largest changes occurring in the development of executive functions (i.e., abstract thinking, decision making and planning, and response inhibition (Yurgelun-Todd, 2007). This potentially reduces the ability of the adolescent athlete to efficiently and effectively express and reproduce perceptions associated with recovery and stress (Steinberg, 2005). Although daily external and internal training loads were slightly lower in professional soccer players, evidence suggests that academy soccer players do not achieve the absolute intensities completed by elite adult soccer players (Malone et al., 2015a). Our results suggest that training load has only a trivial to small impact on athlete-reported levels of stress and recovery in youth soccer players, but more research from different age groups and in turn biological maturity is needed to confirm this finding.

Another interesting finding of this study was that whilst we observed some moderate within-player associations between accumulated training load indicators and changes in CMJ parameters, magnitudes of these observed changes were mostly trivial to small. Similar to previous research (Noon et al., 2018; Sawczuk et al., 2018b; Fitzpatrick et al., 2019) CMJ jump height did not show substantial changes and associations with training load indicators supporting the notion that jump height is a poor parameter to assess responses to load. It has therefore been suggested to examine the responsiveness of parameters reflecting the kinematics and kinetics of the CMJ (Gathercole et al., 2015). However, we observed only small to moderate associations of accumulated training load and changes in most eccentric and concentric kinematic and kinetic parameters. In particular, increased training load was associated with moderate decreases in concentric impulse and somewhat contradictory small to moderate increases in force at zero velocity and rate of force development, respectively. However, force at zero velocity and rate of force development were, on average, both impaired after the 2 days of load accumulation. While it is difficult to ascertain the underlying mechanisms of these findings a reasonable explanation of this might relate to the impaired contractile function (i.e., force capacity, blood flow) and muscle activation (i.e., voluntary activation, neuromuscular propagation) (Enoka and Duchateau, 2016) as a result of the inflammatory process after high-eccentric loading which typically peaks between 24 and 48 h after a training stimulus (Nédélec et al., 2012). In addition, altered stretch-reflex sensitivity and muscle-tendon stiffness have been reported after eccentric loading protocols resulting in reduced force and power production (Nicol et al., 2006). This ultimately reduces mechanical efficiency resulting in altered kinematics and kinetics, particularly for parameters of the CMJ related to the eccentric phase (Byrne et al., 2004). Taken together, the small to moderate associations and changes in CMJ parameters observed in this and previous studies (Sawczuk et al., 2018a; Fitzpatrick et al., 2019; Norris et al., 2021) highlight the difficulty and complexity of assessing neuromuscular responses to load. In addition, despite small to moderate standardized changes, confidence intervals of the absolute changes were rather large and mean absolute changes in CMJ parameters were smaller than the typical day-to-day variability observed in a recent between-day reliability study (Ruf et al., 2021b) questioning their usefulness to detect small changes that are beyond the naturally evident variability of these parameters in high-level adolescent soccer players. As such, the observed changes in CMJ parameters are likely underpowered given the variability in the data failing to observe detectable changes that are beyond the typically observed day-to-day-variability. Future research might therefore look into employing training load indicators measuring more accurately the external and internal neuromuscular loads to ascertain the potential responsiveness of CMJ parameters in relation to periods of increased neuromuscular training loads. In this context, both short- (e.g., training camp) and long-term periods (e.g., pre-season) are of interest to evaluate the acute and chronic responsiveness of the CMJ in particular and measurement instruments in general.

Finally, we observed a moderate decrease in HRex and small increase in HRR60s after two days of load accumulation. While the observed change in HRex (~-2.8%) was substantially greater than the reported day-to-day variations previously reported within similar populations (typical error of measurement: ~1.5%), day-to-day variability for HRR60s (typical error of measurement: 7–16%) exceeded by far the observed change in our study (2.1%) (Rabbani et al., 2017; Doncaster et al., 2019; Ruf et al., 2021b). Only small changes in HRR60s have also been previously reported despite simultaneous substantial decreases in HRex during an 11 day in-season camp in the heat (Buchheit et al., 2011). Similarly, HRex was substantially reduced after several consecutive training days across a 12-week intensified preparatory period in elite Badminton players (Schneider et al., 2020). In addition, we observed a moderate correlation between changes in HRex and total distance. This is consistent with previous observational research from training camps in senior Gaelic Football and soccer, whereby daily changes in HRex were strongly correlated with changes in training load (Buchheit et al., 2013; Malone et al., 2017). The stronger correlations observed in these studies likely reflect the greater day-to-day fluctuations in training load and in turn greater range of changes in HRex. Importantly, while a decreased HRex has also been shown to be associated with chronic improved cardiorespiratory fitness after several weeks (e.g., Buchheit et al., 2012; Altmann et al., 2021), in the context of short-term responses to load, decreases in HRex are likely the result of exercise-induced increases in plasma volume (Schneider et al., 2020). In addition, decreased HRex has been shown to be associated with changes in cardiac autonomic nervous system such as lower sympathetic and higher parasympathetic activity, reduced catecholamine tissue responsiveness and adrenergic receptor activity (Buchheit et al., 2009; Meeusen et al., 2013). Similar to HRex, the strongest correlation for HRR60s with moderate magnitude was observed with total distance. Post-exercise heart rate recovery generally reflects meta-boreflex activity, which partly influences parasympathetic reactivation and sympathetic withdrawal during the initial phase of recovery (Borresen and Lambert, 2008; Buchheit, 2014). This suggests that even greater accumulated training loads are required to elicit substantial changes in the autonomic nervous system and in turn HRR60s after short periods of load accumulation. Our and previous findings suggest that using heart rate during exercise should be preferred to heart rate recovery after submaximal runs to monitor acute cardiorespiratory responses to load.

Limitations of the current study also need to be acknowledged. Although our training load indicators matched the commonly used ones (Nosek et al., 2021) it cannot be ruled out that either (1) training load indicators are unable to provide an accurate estimate of the mechanical demands to reflect subsequent acute psycho-physiological responses (Vanrenterghem et al., 2017), (2) the psycho-physiological response measurement instruments lack responsiveness to fluctuations to training load or (3) the fatiguing period was not high enough to elicit substantial psycho-physiological responses. Further, given the applied nature of the study design, chronic responses resulting from supercompensation effects across the study period were not considered. In addition, physical capacities (e.g., aerobic and anaerobic endurance, strength and power) in combination with other individual characteristics (e.g., biological maturation status) may act as a moderator in the acute responses to load and should therefore warrant consideration when interpreting the responsiveness of the investigated measurement instruments. Further, as training load was not controlled and documented on the day off (i.e., Thursday), players might have engaged in non-football specific physical activity impacting the measurement after the rest day. Finally, data were collected only on a small sample from a single age group of one club as a result of the applied nature of this study which may not be representative to other youth athletes of different biological maturity or from other clubs.

### Practical Application

This study provides practitioners with a better understanding of the relationships between indicators of training load and common measurement instruments to quantify acute responses to load in adolescent soccer players. Our findings suggest that the limited responsiveness of athlete-reported questionnaires and CMJ parameters means that these measurement instruments are unlikely to provide insight into the acute psycho-physiological responses to load. As such, practitioners utilizing athlete-reported questionnaires and CMJ parameters to quantify acute psycho-physiological responses to load should do so with caution while exploring other measurement instruments to provide more nuanced insights into the constructs of fatigue and recovery. In contrast, HRex during a submaximal run reflects acute responses of the cardiorespiratory system to load and might therefore be used by practitioners to manage training load or program adequate recovery. However, changes in HRex need to be interpreted within the context of the training program as acute negative and chronic positive cardiorespiratory changes follow the same pattern.

## Conclusion

Taken together, our results suggest that most of the investigated measurement instruments to assess acute psycho-physiological responses have limited short-term responsiveness to training load. As such, these measures provide limited information for practitioners when evaluating the psycho-physiological response of adolescent soccer players to training load. Future studies should investigate the chronic responsiveness of measurement instruments for adolescents of varying maturity status in order to better understand the usefulness of such parameters to manage subsequent training load and recovery more effectively.

## Data Availability Statement

Due to club policy data cannot be shared. However, we created a detailed outline of the statistical process (see Supplementary File) providing a detailed overview of our approach to analyse the data.

## Ethics Statement

The studies involving human participants were reviewed and approved by Ethics Committee of the Saarland University, Germany. Written informed consent to participate in this study was provided by the participants' legal guardian/next of kin.

## Author Contributions

LR, BD, PE, SS, and TM contributed to the design and conceptualization of the study, to the analysis and interpretation of the results, and to the writing of the manuscript. All authors made substantial contributions to the manuscript and critically revised and reviewed the manuscript and approved the submitted version. All authors contributed to the article and approved the submitted version.

## Conflict of Interest

The authors declare that the research was conducted in the absence of any commercial or financial relationships that could be construed as a potential conflict of interest.

## Publisher's Note

All claims expressed in this article are solely those of the authors and do not necessarily represent those of their affiliated organizations, or those of the publisher, the editors and the reviewers. Any product that may be evaluated in this article, or claim that may be made by its manufacturer, is not guaranteed or endorsed by the publisher.
