# Supplementary material for: Are Measurement Instruments Responsive to Assess Acute Responses to Load in High-Level Youth Soccer Players?
Source: Front Sports Act Living. 2022 Jul 1;4:879858. doi: 10.3389/fspor.2022.879858 (PMC9283776; doi:10.3389/fspor.2022.879858)
Supplement: Supplementary file 1 [file Data_Sheet_1.docx]

Supplementary Table 1. Description of all computed counter-movement jump (CMJ) variables

| **CMJ Variable** | **Unit** | **Abbreviation** | **Description** |
| --- | --- | --- | --- |
| Jump Height | cm | JH | The maximum jump height achieved based on vertical take-off velocity: take-off velocity^2^ ÷ 2g. |
| Reactive Strength Index modified | m.s^-1^ | RSImod | Ratio between jump height and total time to take-off. |
| Eccentric Rate of Force Development | N.s^-1^ | RFD | Largest force increase during a 30 ms epoch. |
| Eccentric Impulse | N.s | EccI | Force exerted during eccentric phase multiplied by the time of the eccentric phase. |
| Concentric Impulse | N.s | ConI | Force exerted during concentric phase multiplied by the time of the concentric phase. |
| Eccentric Velocity | m.s^-1^ | EccV | Mean velocity achieved during the eccentric CMJ phase. |
| Concentric Velocity | m.s^-1^ | ConV | Mean velocity achieved during the concentric CMJ phase. |
| Force at Zero Velocity | N | F@0V | Force when velocity is zero (transition from eccentric to concentric). |
| Duration of Eccentric Phase | ms | DurEcc | Time required to perform the eccentric CMJ phase. |
| Duration of Concentric Phase | ms | DurCon | Time required to perform the concentric CMJ phase. |
| Countermovement Depth | cm | CMD | The minimum (i.e. peak negative) displacement when velocity is zero (transition from eccentric to concentric). |

**Example process for statistical analysis**

# packages

library(MASS) #build ordered logistic regression models
library(sjPlot) #create tables for models
library(nlme) # create linear-mixed models
library(rmcorr) # repeated-measures correlation
library(boot) # CI bootstrapping
library(knitr) # create generic tables

# Analysis of SRSS using ordered logistic regression models

## SRSS_Physical_Performance_Capability
### model
odds_KEB_kL <- polr(as.factor(SRSS_Physical_Performance_Capability) ~ as.factor(assessment_time_cycle),
 Hess = TRUE,
 method = c("logistic"),
 data = Data_raw_long_LMM)

### summary
summary(odds_KEB_kL)

tab_model(odds_KEB_kL,
 show.ci = 0.90)

## store table
odds_KEB_kL_table <- coef(summary(odds_KEB_kL))

## calculate and store p values
odds_KEB_kL_p <- pnorm(abs(odds_KEB_kL_table[, "t value"]), lower.tail = FALSE)*2

## combine tables
cbind(odds_KEB_kL_table, "p value" = odds_KEB_kL_p)

## calculate 90% CI
odds_KEB_kL_CI <- confint(odds_KEB_kL, level = 0.90)

## odds ratio
exp(coef(odds_KEB_kL))

## odds ratio with 90% CI
exp(cbind(OR = coef(odds_KEB_kL), odds_KEB_kL_CI))

# Analysis of parameters of the CMJ and submaximl run using linear mixed models

# Jump_Height
## model
lme_JH <- lme ( Jump_Height ~ assessment_time_cycle,
 random = (~ assessment_time_cycle | player_ID),
 weights = varIdent(form = ~ 1 | assessment_time_cycle),
 corr = corAR1(form = ~ 1 | player_ID),
 data = Data_raw_long_LMM)

## summary
summary(lme_JH)

## table
tab_model(lme_JH,
 show.ci = 0.90)

# phi
lme_JH$modelStruct$corStruct

# Standardised mean differences for parameters of the CMJ and submaximl run

# set seed
set.seed(1312)

## Jump_Height
### Subset variables, create dataframe, add a change score column
SMD_df_JH <- data.frame(Data_raw_long_0$`Jump Height`,
 Data_raw_long_1$`Jump Height`) %>%
 drop_na() %>%
 dplyr::rename("pre" = "Data_raw_long_0..Jump.Height.",
 "post" = "Data_raw_long_1..Jump.Height.") %>%
 mutate(diff = post-pre)

### Function
SMD_function_JH <- function(data, indices) {
 d <- data[indices,]
 n_pre <- length(d$pre)
 n_post <- length(d$post)
 sd_pre <- sd(d$pre, na.rm = TRUE)
 sd_post <- sd(d$post, na.rm = TRUE)
 sd_pooled <- sqrt(((n_pre-1)*sd_pre^2 + (n_post-1)*sd_post^2) / (n_pre+n_post-2))
 m_diff <- mean(d$diff)
 ds <- m_diff/sd_pooled
 result <- c(ds)
}

### Boot function
SMD_boot_JH <- boot(SMD_df_JH, SMD_function_JH, R = 10000)


### Extract the values
SMD_CL_JH = boot.ci(SMD_boot_JH, conf = 0.90, type="bca")

### Create lists of ES and CL
SMD_names_JH = c("JH")
SMD_ES_JH = round(c(SMD_CL_JH$t0),2)
SMD_LL_JH = round(c(SMD_CL_JH$bca[4]),2)
SMD_UL_JH = round(c(SMD_CL_JH$bca[5]),2)

### create data frame with ES and CI
SMD_ES_CI_JH = data.frame(SMD_names_JH, SMD_ES_JH, SMD_LL_JH, SMD_UL_JH) %>%
 dplyr::rename("Parameter" = SMD_names_JH,
 "Effect Size" = SMD_ES_JH,
 "Lower Confidence Limit" = SMD_LL_JH,
 "Upper Confidence Limit" = SMD_UL_JH)

### final table with ES and CI for parameter
SMD_ES_CI_JH

# Repeated-measures correlation for parameters of the SRSS, CMJ and submaximal run

# Jump Height
## total distance
rm_Total_JH_1_2 <- rmcorr(dataset = Data_delta_long,
 participant = `ID`,
 measure1 = `Total day-1&2 post measurement`,
 measure2 = `Jump Height`,
 CI.level = 0.90,
 CIs = c("analytic"),
 nreps = 10000,
 bstrap.out = TRUE)
rm_Total_JH_1_2

## high speed distance
rm_High_Speed_JH_1_2 <- rmcorr(dataset = Data_delta_long,
 participant = `ID`,
 measure1 = `High-Speed Distance day-1&2 post measurement`,
 measure2 = `Jump Height`,
 CI.level = 0.90,
 CIs = c("analytic"),
 nreps = 10000,
 bstrap.out = TRUE)
rm_High_Speed_JH_1_2

## sRPE
rm_sRPE_JH_1_2 <- rmcorr(dataset = Data_delta_long,
 participant = `ID`,
 measure1 = `sRPE day-1&2 post measurement`,
 measure2 = `Jump Height`,
 CI.level = 0.90,
 CIs = c("analytic"),
 nreps = 10000,
 bstrap.out = TRUE)
rm_sRPE_JH_1_2
